# Peptide-Based Technologies to Alter Adenoviral Vector Tropism: Ways and Means for Systemic Treatment of Cancer

**DOI:** 10.3390/v6041540

**Published:** 2014-04-02

**Authors:** Julia Reetz, Ottmar Herchenröder, Brigitte M. Pützer

**Affiliations:** Institute of Experimental Gene Therapy and Cancer Research, Rostock University Medical Center, Schillingallee 69, Rostock 18057, Germany; E-Mails: julia.reetz@uni-rostock.de (J.R.); ottmar.herchenröder@uni-rostock.de (O.H.)

**Keywords:** adaptor protein, adenovirus, chemical modification, gene therapy, peptide ligand, targeting strategy, vector

## Abstract

Due to the fundamental progress in elucidating the molecular mechanisms of human diseases and the arrival of the post-genomic era, increasing numbers of therapeutic genes and cellular targets are available for gene therapy. Meanwhile, the most important challenge is to develop gene delivery vectors with high efficiency through target cell selectivity, in particular under *in situ* conditions. The most widely used vector system to transduce cells is based on adenovirus (Ad). Recent endeavors in the development of selective Ad vectors that target cells or tissues of interest and spare the alteration of all others have focused on the modification of the virus broad natural tropism. A popular way of Ad targeting is achieved by directing the vector towards distinct cellular receptors. Redirecting can be accomplished by linking custom-made peptides with specific affinity to cellular surface proteins via genetic integration, chemical coupling or bridging with dual-specific adapter molecules. Ideally, targeted vectors are incapable of entering cells via their native receptors. Such altered vectors offer new opportunities to delineate functional genomics in a natural environment and may enable efficient systemic therapeutic approaches. This review provides a summary of current state-of-the-art techniques to specifically target adenovirus-based gene delivery vectors.

## 1. Introduction

**Figure 1:**
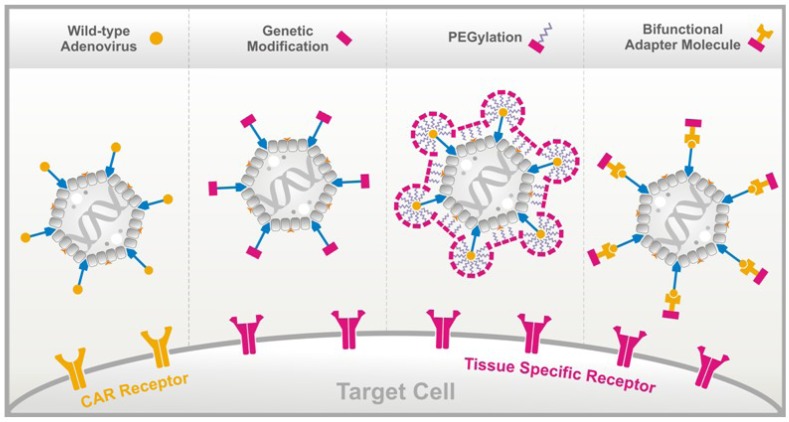
Methods to alter adenovirus tropism. (**A**) Wild-type adenovirus enters target cells after binding the coxsackie-adenovirus receptor (CAR), an entity present on a wide number of cell types. Ablation of CAR binding and re-directing adenovirus-derived vectors towards the cells of choice by means of specific peptides can be achieved by: (**B**) genetically integrating the peptide into the fiber knob; (**C**) chemically coating the vector particle with bi-specific polyethylene glycol (PEG); or (**D**) through bridging by means of a bifunctional adapter molecule.

Adenoviruses (Ads) are common pathogens in humans and other vertebrate hosts. Adenoviridae are typically divided into four genera [1] and subdivided into different species or subgroups denoted A to G [2]. After the first human adenovirus was isolated in 1953 by Rowe [3], to date, a total of 57 different serotypes have been identified and were subclassified on the basis of parameters regarding classical standard methods, such as the rat hemagglutination assay and, later, genomic analyses [4]. Adenovirus particles consist of a non-enveloped icosahedral capsid that carries a single continuous molecule of double-stranded DNA as the viral genome. For most serotypes, Ad entry into host cells is initiated by an interaction with the knob, the outmost bulging tip of the virion’s fibers. These trimeric rigid structural elements protrude from each of the capsid’s twelve vertices, the penton bases, where they are non-covalently anchored. The fiber knobs mediate binding to the cell surface’s receptor moieties, such as the coxsackie adenovirus receptor (CAR), the major high-affinity receptor for many adenoviruses (Figure 1), which is a 46-kDa transmembrane protein in the immunoglobulin superfamily [5,6,7]. The subsequent internalization of the viral particle depends on a secondary interaction of an Arginine-Glycine-Aspartate (RGD) protein structure on the Ad’s penton bases with αVβ3 or αVβ5 integrins on the cell surface [8]. Due to the abundance of CAR in many tissues, adenoviruses display a broad tropism and infect a wide spectrum of cells [9,10,11]. This characteristic drew an early focus on this virus family as masterpieces for gene therapeutic approaches. Unlike other viral vector systems, Ad virions have a high packaging capacity and easily replicate to high titers. Moreover, Ad-derived vectors maintain high stability *in vivo* and transduce both dividing and non-dividing cells [12]. They predominantly persist as episomal DNA molecules with an extremely low frequency of integration into the host genome [13], and thereby, insertional mutagenesis does not occur. These properties make Ad-based vectors particularly attractive for gene therapy applications, where temporary gene expression is desired or preferred over permanent genetic modifications. The most commonly utilized Ad vectors for gene therapy are derived from adenovirus type 5 (Ad5) in the subgroup C of human adenoviruses, due to their apathogenicity and non-oncogenic properties [9,14].

Tissue tropism of adenoviral vectors is greatly influenced by the viral serotype and receptor presence and density, depending on a cell type’s provenance [8,9,15,16,17]. The best studied and most widely used Ad5-derived vectors exclusively bind to CAR. However, in the case of Ads derived from Serotype 41, a member of Subgroup F, only one of its two distinct types of fiber can recognize CAR [2,18]. Some serotypes enter host cells via other receptors, such as CD46, desmoglein 2, CD80, or CD86 or the sialic acid moiety (reviewed by Sharma [19]). Altogether, Ad5 vectors are efficient vehicles for delivering foreign genes into target cells *in vitro* [20], and due to their favorable attributes, they are utilized in more than one-quarter of all human gene therapy trials and have a proven safe clinical profile [21].

However, the utilization of adenoviral vectors as targeted gene delivery systems is hampered by their tropism for a broad range of cells and tissues [22]. Once a system is required that exclusively alters a single cellular compartment or a particular kind of tissue that spares all other cells and tissues from transduction, receptor alteration of vector particles becomes essential. Another major obstacle after systemic adenoviral vector administration is that 80% of circulating particles are sequestered in the liver after an interaction with coagulation factors [23,24], and thereby, most particles may not reach the tissue to be addressed. Doronin and co-workers demonstrate that after the virus is recognized by the coagulation system, the immune system is activated, and in turn, an acute inflammatory response is initiated [25,26,27,28,29,30]. In addition, regarding Ad transport in the bloodstream, Duffy and colleagues [31] identified a number of small molecules capable of efficiently blocking the intracellular virus transport independently of factor X-associated inactivation. Considering all these drawbacks, a selective gene transfer by wild-type adenoviral vectors imposes an increased risk of toxicity, due to Ad vector dissemination to non-targeted cells, even if the vectors are administered close to or directly into the tissue of interest. Other undesired side effects of systemic virus administration are virus-associated immunogenic toxicity, thrombocytopenia, intense periportal polymorphonuclear lymphocyte infiltration and elevated liver enzyme secretion [22,32,33].

The reverse obstacle is the question of how to reach cell types refractory to adenoviral infection, due to their lack of or insufficient CAR expression. Such cells include, for example, many cancer cells, as well as hematopoietic and neural stem cells [34]. To achieve gene transfer into those cell types and to ensure efficient integrin receptor-mediated virus uptake, extremely high vector doses are required. High vector doses in turn increase inadvertent side effects, like viral sequestering in Kupffer cells in the liver [35], and once vectors surpass the latter’s binding capacity, hepatocytes will absorb the remaining vector particles. 

The restrictions outlined above can be overcome by strategies to modify the vector’s cellular tropism, as reviewed by Beatty and Curiel [36]. Redirecting vectors towards cells of interest can also enhance the therapeutic potential with increased safety by reduction of immune responses, since simultaneous re- and de-targeting allows lower vector doses to be administered systemically [37,38,39,40]. In this review, we present and discuss three different methods to alter the natural Ad vector tropism by: (i) genetic integration of peptide sequences into the fiber; (ii) chemical peptide conjugation via polyethylene glycol (PEG); and (iii) bridging the vector and cell with bispecific adapter molecules. The advantages and benefits, as well as restrictions and limitations of these technological approaches are described and debated below. The initial considerations towards targeting, however, relate to the identification of suitable moieties on the plasma membrane of the cells or the tissue to be addressed that fulfil the following characteristics: singularity, abundance and affinity. 

## 2. Screening for Cell-Specific Ligands

The ultimate gene “taxi” for systemic gene therapy purposes should exclusively recognize the cells to be treated and leave all others unaffected. Directed gene delivery can be achieved by addressing selective moieties on the cells of interest. Peptides possess appropriate properties to serve as targeting agents and are valid alternatives to antibody-based targeting approaches, since unique cellular receptors are often unknown. 

The simplest way to design a specific binding peptide for a receptor is to start with the structural data of the binding parts [41]. The solution is almost the problem if the structural data are not available. To solve this, the phage display is a frequently used technique to determine specific binding peptides [42]. We and others have used the phage display technology to screen for and to identify tissue- or cell-specific ligands in culture systems and animal models. As early as 1990, researchers constructed an epitope library that yielded a mixture of filamentous phage clones, each displaying one peptide sequence on the virion surface [43]. After the interaction of the phage and the specific binding partner, the expansion of the phage comprehends several rounds of infection followed by selection. The display of polypeptide repertoires on the surface of phages, together with the efficient enrichment and amplification of the desired binding specificities was then shown to be a valuable route towards isolation of unique peptides that can act as vehicles for targeting applications [44,45,46,47,48,49,50]. The phage display technique identifies peptides in a range from eight to 12 amino acids [42]. The technique was successfully employed to acquire peptides that specifically recognize human embryonic progenitor cells [51] and bind normal and diseased tissue, like vascular endothelium [52,53,54,55,56], lymphatic vessels [57], kidney tubules [58] and several others [50,59,60,61,62,63,64]. Furthermore, the lack of gene transfer systems that are potent in selectively targeting cancer tissues prompted the search for tumor-specific peptide molecules for yet unknown tumor-associated receptors [65,66,67,68]. Many novel peptides homing to angiogenic vessels showed cross-affinity with several tumor types [56]. In this regard, we conducted biopanning on human medullary thyroid carcinoma (MTC) cells *in vitro* and transplanted tumor xenografts *in vivo*. MTC, which is caused by dominant activating mutations in the RET proto-oncogene encoding a transmembrane tyrosine kinase receptor, is characterized by aggressive growth and early metastasis and, therefore, provides a perfect model for targeting disseminated cancer cells [69]. The selected phages bound with highest specificity to and were internalized by these tumor cells in culture and after systemic injection into nude mice [70]. The same 7-mer cyclic phage peptide library was injected into the tail vein of RET oncogene transgenic mice carrying bilateral orthotopic tumors in their thyroid glands [71]. The ligand, which also binds efficiently to human MTC cells was covalently linked to the Ad capsid that carried a RET inhibitor as therapeutic gene. Systemic delivery of this peptide-tagged Ad vector led to the substantial growth reduction of orthotopic and disseminated xenograft tumors, while the interaction with other organs, such as the liver, was largely abolished [39]. This precedent opens a road towards using peptide-mediated adenoviral gene transfer to achieve an efficient and selective antitumoral response, even at the stage of metastatic disease. Beyond that, other researchers took the first step in developing a molecular map of the human vasculature by screening a peptide library in patients [72,73,74]. Rangel and co-workers [75] developed a novel technology that enables receptor-independent phage particle entry into mammalian cells. Phage particles provide a unique discovery platform for combinatorial intracellular targeting of organelle ligands along with their corresponding receptors and for fingerprinting functional protein domains in living cells [76].

An alternative approach that aims at new molecules with high affinity, adequate specificity and suitable pharmacokinetic properties for *in vivo* applications is represented by single-stranded nucleic acid ligands, termed aptamers. Aptamers are isolated by Systematic Evolution of Ligands by Exponential Enrichment (SELEX) technology. Applying this technology against whole-living cells *in vitro* or *in vivo* allows direct selection of aptamers even against rare antigens without prior purification of membrane-bound targets, access to membrane proteins in their native conformation and identification of targets related to a specific phenotype. Their thermal stability, low cost, unlimited applications and high binding affinity to disease-associated proteins or non-protein targets [32,77,78,79] make them attractive, even in clinical trials for the treatment of distinct medical conditions, as reviewed very recently [80,81,82]. The potential of aptamers as a valid tumor targeting gene delivery system with high transduction efficiency was summarized by [83,84]. In this perspective, an innovative step towards targeted therapies would certainly be a combination of technologies, the cell-specific aptamers and adenoviral vectors. 

## 3. Methods to Alter the Natural Ad Vector Tropism

### 3.1. Genetic Fiber Engineering

To increase the selectivity of adenovirus for target tissues, novel approaches in Ad vector design exploit the concept of tissue-specific expression of therapeutic transgenes or virus replication. The development of genetically targeted vectors has focused on the fiber, since it is the major determinant of adenovirus tropism. It is a homotrimeric protein characterized by a domain organization with an N-terminal tail domain anchoring the fiber in the Ad capsid, a C-terminal globular domain, termed the knob, that mediates binding to CAR and a central shaft domain extending the knob away from the virion. Mutations of the fiber knob *per se* significantly reduce the transduction efficiency of CAR-positive cells by Ad vectors [85,86,87]. Different strategies of adenovirus fiber modification have been employed, like genetic replacement of the fiber or ligand incorporation into the fiber knob. In this regard, the development of a fiber phage display system [65,88] or a fiber-shuttle library for the adenoviral knobs [89] provide tools to alter Ad vector binding specificity. 

Several studies narrowed down the insertion positions for targeting peptides within the fiber knob to two locations where the vector system as such tolerates the genetic alterations without structural impairment. The sites of choice for targeting ligand incorporation are the fiber knob’s HI loop, which connects the ß-strands H and I, and the C-terminus of the protein [86,90,91,92,93,94,95,96]. These findings indicated that ligands whose sizes exceed 25 to 30 amino acid residues cannot be configured into the carboxy-terminus of the fiber, as they destabilize the fiber structure [85] and, thus, limit the range of potential ligand candidates to short peptides. The structural properties of the HI loop of the Ad fiber, however, favor the insertion of larger ligands and expand the size of potential targeting moieties. When testing the resilience of fiber modification, Belousova and co-workers incrementally increased the size of the peptides integrated in the HI loop and generated Ad vectors with fiber inserts ranging from 13 to 83 amino acid residues [97]. The authors concluded that the incorporation of heterologous sequences in the examined size ranges was essentially tolerated without a negative impact or compromising the production yield or infectivity of the vectors. HI-loop incorporation of rather short 7- and 9-mer peptides was performed to transduce CAR-deficient primary tumor cells, such as ovarian cancer cells, vascular endothelia, vascular smooth muscle cells and brain microcapillary endothelia in culture [5,37,40,50,90,98,99]. The last step of Ad entry into target cells depends on the interaction between RGD motif at the penton base protein and the host cell integrins [100]. Ad vectors containing this RGD peptide in the HI loop of the fiber showed higher yields of gene transfer than vectors containing the identical peptide attached at the fiber‘s C-terminus, due to the easy access to the receptor [39,101,102]. Many groups transduced effectively different types of tumor cells by inserting this RGD motif into the HI loop of the Ad fiber *in vitro* [103] and *in vivo* [104,105]. Rojas *et al*. [106] improved systemic antitumor therapy with oncolytic adenoviruses by replacing the fiber shaft heparin sulfate glycosaminoglycan-binding domain with RGD in order to achieve simultaneously liver de- and tumor re-targeting. 

Each fiber knob monomer forms an eight-stranded antiparallel ß-sandwich structure. The ß-strands are connected with turns and loops. To further reduce the transduction efficiency of Ad vectors to CAR-positive cells, mutations of the AB, DE or FG loop of the fiber knob have been reported [55,107,108]. Important is, however, that in addition to the genetic fiber modification, this secondary minor interaction of the RGD motif at the penton base with the αv-integrin receptor must be depleted, as well [109], to completely ablate the Ad vector from the native binding moieties. Whereas the dual mutation markedly reduces the retention of the vector in the liver [87,92], single mutations in the fiber knob or penton base did not alter the biodistribution of adenoviral vectors injected into mice [108,110,111,112,113]. In order to use Ads in cancer gene therapy, gene transduction to cancer cells is limited by the weak expression of the CAR receptor on these cells, reviewed by [101]. Magnusson and co-workers [114] efficiently transduced human ovarian and breast cancer cell lines with a vector that carried the human epidermal growth factor receptor 2 into the HI loop of a CAR ablated fiber knob. Later, the group generated a vector with dual specificity by incorporating the HER2/neu- (ZH) and Taq polymerase-binding (ZT) sequence at different positions within the HI-loop. Receptor-binding studies revealed that ZT in the first position and ZH in the second position bound to both receptors, whereas the reverse order of both motifs was devoid of binding to HER2/neu [115]. Subsequently, these researchers designed a vector to transduce efficiently human epidermal growth factor receptor 2-presenting cell lines, by altering the RGD motif to EGD (Glu-Gly-Asp) and substituting the KKTK motif, residues 91–94, in the third shaft repeat, to RKSK (Arg-Lys-Ser-Lys). This new vector in the end gained the ability to efficiently infect prostate cancer cells *in vitro* [114].

Genetic modification also covers the replacement of the entire fiber or just the knob domain with that derived from other adenovirus serotypes [116]. Belousova *et al*. [117] targeted an Ad vector with bacteriophage T4 fibritin to the CD40 receptor. The tropism was modified by incorporating into the virion capsid a recombinant protein comprising structural domains of the Ad Serotype 5 fiber, phage T4 fibritin and the human CD40 ligand. The authors achieved specific gene delivery in monocyte-derived dendritic cells (DC) *in vivo*. In a pilot vaccination study, Thacker and colleagues [32] targeted successfully DC cells in an *in vivo* canine model by integrating the CD40 ligand into the fiber knob. The same group reported later that Ad vectors bound to the CD40 ligand failed to infect integrin-deficient canine lymphoma cells. This study demonstrates that the lack of virus internalization signals can impair targeting approaches [118]. 

Yu and co-workers [119] reported on modifying the Ad5 hexon protein by inserting the protein transduction domain from the HIV-1 Tat protein. The resulting viral vector showed significantly higher transduction efficiency on many tumor cells compared to the parental vector. In the next step, this group increased the infection efficiency of human primary cell types further after swapping wild-type Ad5 fiber against a Serotype 35 fiber specific for the CD46 receptor, which is upregulated in a number of tumors. This surface modified Ad vector was developed to transduce otherwise difficult-to-transduce cells in basic, pre-clinical and clinical research [120,121]. Another strategy to reroute adenoviral vectors from normal towards cancer tissue is the utilization of recombinant adenovirus. Sova *et al*. [122] constructed a capsid-modified adenovirus that specifically replicates in tumor cells and expresses the TNF-related apoptosis-inducing ligand (TRAIL). Their Ad capsid contains the Serotype 35-derived short-shafted fibers, which recognize the CD46 receptor, a surface marker often upregulated in malignant tumors. In combination with TRAIL, expression of this oncolytic vector induces apoptosis in tumor cell lines derived from human colorectal, lung, prostate and liver cancer. Both the cell culture and xenograft tumor models tested in these experiments showed efficient intratumoral spread of the virus. Another group designed Ad vectors presenting the short fibers of Ad41 as a ligand insertion tool, achieving higher infection efficiency when compared to viruses presenting the same ligand incorporated into another part of the fiber [123]. Even an enhanced transduction efficiency of recombinant adenovirus Type 5 vectors with Serotype 35 fibers (f35) was observed by Matsui and co-workers [124]. Using a feasible *in vitro* ligation, the group incorporated two copies of the RGD peptide in two different loops of the f35 knob and observed high infection efficiencies in CD46-positive cells.

Overall and despite these positive results, genetic modifications to the native Ad5 fiber knob have proven to be a laborious technical cast with effectiveness that is hard to calculate. The repertoire of incorporable ligands to yield functional retargeted vectors for gene therapy is restricted to a small number of peptides that do not impair correct folding and assembly of the fiber trimer [125]. A general limitation of this approach is the necessity to tediously re-engineer a given Ad vector for every further target cell. 

### 3.2. Chemical Peptide Conjugation via PEGylation

An option to covalently link targeting peptides to otherwise unaltered Ad vector particles is by means of bispecific non-toxic spacer molecules. Here, polyethylene glycol (PEG) is a hydrophilic linear polymer widely used in galenic formulations, as reviewed by [126]. Optimized transduction by targeted Ad vectors can be accomplished by linking cell-specific peptides, antibodies or antigens to the particle’s surface by a chemical process called PEGylation [127]. PEG forms a covalent bridge between the proteins of the virion’s surface and the targeting molecule of choice, resulting in a vector coated all over with the desired ligand. This approach of redirecting viral vectors does not require genetic modification, including the efforts to ablate the native tropism. Additional benefits of PEGylated vectors are reduced immunogenicity [128,129,130,131], fewer hepatotoxic side effects [132], less cytokine secretion and the prolongation of the vector plasma half-life [133]. 

In the meantime, such PEG-driven Ad vector modifications have been adapted in a good number of targeting approaches [133,134,135,136,137,138,139]. The success of such approaches might depend on the ligand length. Romanczuk and colleagues [135] were the first to link biologically selected peptides to Ads surface via PEGylation. For instance, coupling of a short RGD motif to the tip of PEG has shown both high *in vitro* transduction efficiency [140,141,142,143] and an improvement of systemic gene delivery [132,144]. In an ovarian cancer targeting approach, full-length fibroblast growth factor 2 (FGF2) was linked to an Ad vector by PEGylation. This vector mediated increased transgene expression in tumor tissue and reduced localization of adenovirus to non-target cells when compared to unmodified Ad [140]. To silence the proinflammatory activation status of endothelial cells, Kuldo and co-workers [145] demonstrated the potential of an E-selectin targeted Ad vector to deliver a therapeutic transgene into microvascular endothelial cells in inflammation and downregulate the endothelial adhesion molecule. As demonstrated by Kim *et al*. [146], PEGylated Ad that recognizes Her2/neu receptor-positive cancer cells showed longer circulation times than the unmodified control and decreased the level of neutralizing antibodies. These observations could raise positive expectations for future therapeutic applications of PEGylated vectors against late-stage cancer diseases. Exploring the suitability of PEGylated Ad vectors to address metastatic tumors, a dual cancer-specific strategy was described using this technology for transductional targeting with transgene expression under control of the telomere reverse transcriptase promoter (TERT) for transcriptional targeting [147]. With regard to the conclusion that the molecular weight of PEG and the PEG modification ratio significantly affects the characteristics of conjugates [148], Eto and colleagues [136] optimized adenovirus PEGylation in a way that after systemic administration of PEGylated adenoviral vector expressing tumor necrosis factor-alpha an antibody reduction against Ad, and an increased therapeutic response against metastatic cancer was observed. Yao and co-workers demonstrated in a quite elegant experiment that the CGKRK (Cys-Gly-Lys-Arg-Lys) peptide conjugated to Ad with PEG was highly selective and yielded good gene expression in tumor and tumor vasculatures after systemic administration. At once, their results indicate an important aspect to consider when working with Ad cross-linked with PEG. The appropriate ratio between PEG and targeting ligand concentration is crucial to achieve specific tissue transduction [149,150]. As described above, the latest success in the treatment of disseminated tumors was made by injecting a low dose (10e^8^ plaque forming units (pfu)/animal) of Ad vector encoding RET oncogene inhibitor coated with MTC-specific 7-mer peptide via PEG into the tail vein, which led to the regression of multiple orthotopic and xenograft tumors in mouse models [39]. The same Ad-PEGylation approach using a short artificial peptide selected by phage display, which in this case, specifically binds neural precursor cells isolated from the hippocampus of adult mice, was highly effective after injecting the vector into the brain [40]. Such tools could eventually serve to exclusively manipulate neural stem cells either by direct injection in the brain or systemic vector application with the potential as a delivery system for therapeutic genes to treat various central nervous system disorders. 

Another chemical Ad modification using diblock copolypeptides as an alternative for PEG was first described by Jiang and co-workers [151]. Copolypeptides are well-defined polypeptide sequences [152] providing efficient non-covalent Ad vector modification altering the native Ad tropism with further potential application in targeting cancer metastasis.

### 3.3. Bifunctional Non-Covalently Linked Adapter Molecules

Another way for re-directing and widening Ad vector tropism is the application of bispecific antibody fragments or other bifunctional adapter molecules composed of an anti-fiber antibody portion and a binding component specific for a cell-specific receptor or secondary antibody conjugated with a peptide moiety against specific cell surface antigens. Since CAR does not play any role in virus internalization, the Ad fiber knob’s CAR binding domain accessibility is dispensable and, therefore, the candidate of choice to link heterologous binding sites, for instance a bispecific adapter molecule. A fully studded Ad vector particle with a bridging molecule prevents any interaction with CAR and, thus, ablates Ads native tropism [153,154,155]. 

In the initial demonstration of CAR-independent targeting, a conjugate consisting of folate and a fragment derived from an anti-fiber antibody was used as a recombinant protein construct to bind the Ad fiber, as well as the target, the folate receptor, which is overexpressed on the surface of a variety of malignant cells [156]. In a similar strategy, a conjugated FGF was used to target ovarian carcinoma cells [157]. The approach reached a clinical trial, where FGF2-conjugated Ad vector expressing human herpes simplex virus thymidine kinase was applied in patients [158]. Reynolds *et al*. [159] succeeded in targeting pulmonary endothelial cells *in vivo* by intravenous injection of Ad vectors complexed with a bispecific antibody against the Ad fiber knob and the angiotensin-converting enzyme. 

In light of the development of new therapeutic strategies for diseases in which angiogenesis plays an important role and considering that physiological barriers for high molecular weight components prevent the transduction of the majority of tumor cells, vascular targeting became a worthwhile approach in cancer gene therapy [160]. Targeting of adenovirus to endothelial cells by a bispecific fusion protein directed against the human endoglin CD105 receptor for antivascular cancer gene therapy was published by Nettelbeck and coworkers [161]. In 2004, the same group designed a single-chain adapter molecule that binds the fiber protein and the high molecular weight melanoma-associated antigen. This antigen is widely and specifically expressed on the surface of melanoma cells, and its expression is associated with tumor development and progression [93]. Other bispecific constructs directing Ad fibers to cells were developed for the epidermal growth factor receptor [162,163], endothelial receptors [38] and the lymphocyte antigen 6 complex [164]. A rather elegant approach uses a soluble truncated form of CAR as the virus attachment site fused to human epidermal growth factor (EGF) to direct a vector against cancer cells that express the EGF receptor [165,166,167]. In addition, a number of authors described the adapter-based strategy to target CAR-less dendritic cells as a therapeutic vaccination against cancer or infectious diseases [88,168,169,170,171,172].

Another approach developed by Watkins and colleagues [173] used a construct that encodes a fusion protein derived from a neutralizing anti-adenovirus fiber single-chain antibody, designated S11, fused to a specific peptide ligand directed against cellular receptors, termed the bispecific adapter molecule. Coating virus with this adapter molecule ablates CAR binding and directs the viral particle to the desired cellular receptor. S11 can be produced in eukaryotic, as well as prokaryotic cells. By means of its 6-His-tag, purification and concentration of the fusion protein can be easily performed by nickel-affinity chromatography. This procedure ensures the high yield of pure protein without the loss of activity. Based on the S11 strategy, we intended to specifically transduce *in vitro* and *in vivo* activated hepatic stellate cells (HSCs), whose number is increased in fibrotic livers [41]. Therefore, we picked a peptide derived from nerve growth factor (NGF_p_) with specific affinity for the p75 neurotrophin receptor (p75NTR) present on activated HSCs. Coating the GFP-expressing Ad vector with NGF_p_ was done either via chemical conjugation using bifunctional PEG or, alternatively, by molecular bridging with an S11-based fusion protein specific for viral fiber knob and p75NTR (S11-NGF_p_). After systemic administration of the targeted viral particles, we observed that Ad.GFP-S11-NGF_p_ transduced activated HSCs better than Ad.GFP-PEG-NGF_p_. The latter’s low transduction potential could be explained by an improper ratio between PEG and targeting ligand concentration that prevented successful and specific tissue transduction or due to the ablation of the viral internalization signals by the chemical procedure. These experiments contributed to the development of a targeted gene transfer system to specifically deliver antifibrotic compounds into activated HSCs by systemically applied adenoviral vectors modified by the NGF_p_ ligand. In our study, we demonstrate that adenoviral-mediated targeting of HSCs via p75NTR, concurrently avoiding its binding to hepatocytes, provides a potentially feasible and effective strategy for therapeutic gene delivery to activated HSCs in the liver *in vivo*. Haisma *et al*. [38] observed a selective targeting of Ad5 to the endothelial receptors *in vitro* and obtained viral transgene expression only in tumors infected with adenobody retargeted adenovirus from mice bearing subcutaneous colon carcinoma.

An interesting alternative to the single-chain antibody targeting approach are Ad vectors coated with an adapter molecule based on designed ankyrin repeat proteins (DARPins). DARPins differ from antibodies in size, structure, binding pattern and stability. These properties paired with high-yield, easy production in *E*. *coli* make them promising candidates for targeting purposes. Dreier and co-workers designed an adapter molecule consisting of two DARPin modules fused to each other. One binding site anchors the molecule to the Ad fiber knob, and the other enables the particle to attach to tumor cell markers, like the human epidermal growth factor receptor, the epithelial cell adhesion molecule or the epidermal growth factor receptor. In their work, the authors demonstrate that DARPins are high-affinity adapter molecules that allow efficient gene transfer and are a promising tool to rapidly target Ad vectors against any desired receptor [174,175]. 

The recombinant fusion protein approach offers a number of technical advantages over chemical conjugation, including simplified production in prokaryotic or, preferably, in eukaryotic expression systems, as well as vector purification. In addition, this approach may allow the application of different fusion proteins suitable for retargeting Ad to other receptors, simply by the substitution of the peptide ligand. This procedure offers, according to our experience, the method of choice to retarget Ad vectors. Figure 1 provides a schematic representation of all three strategies used to alter virus tropism. 

## 4. Conclusions

In conclusion, adenoviral vectors have been proven to serve as efficient tools for gene delivery when temporary gene expression is beneficial. The major challenge towards applying the technology remains the development of a target system for specific gene delivery that reaches a high level of efficiency. While genetic fiber manipulation requires tediously re-engineering a given Ad vector and PEGylation causes poor transduction efficiencies due to improper PEG to ligand ratios, as well as RGD ablation, bifunctional adapter molecules seem to be the most promising targeting approach. An expeditious and simple production followed by a broad portfolio of different fusion proteins suitable to retarget Ad by substitution peptide ligands offers a standardized method to retarget vectors for both *in vitro* and/or *in vivo* applications. Moreover, the engineering of bifunctional adaptors may be customized much more easily than fiber modifications and chemical treatments of vector preparations. Increased knowledge of adenovirus biology and powerful techniques to identify new cellular or tissue targets provide the opportunity to develop innovative strategies, which will lead to the selective accumulation and activity of a therapeutic gene or a potent inhibitor of pathogenic genes in the tissue of interest, which may, in future, allow success in the treatment of patients with systemic disease. 
